# The relationship between postnatal depression, sociodemographic factors, levels of partner support, and levels of physical activity

**DOI:** 10.3389/fpsyg.2014.00597

**Published:** 2014-07-14

**Authors:** Maryam Saligheh, Rosanna M. Rooney, Beverley McNamara, Robert T. Kane

**Affiliations:** ^1^School of Psychology and Speech Pathology, Curtin UniversityPerth, WA, Australia; ^2^School of Occupational Therapy and Social Work, Curtin UniversityPerth, WA, Australia

**Keywords:** postnatal depression, partner support, physical activity and wellbeing, sociodemographic factors, psycho-social factors

## Abstract

**Background:** postnatal depression (PND) is defined as a psychological mood disorder that occurs in a mother within 6 weeks of her giving birth. It refers to an episode that causes mood disturbance and it could begin in, or extend into, the postpartum period. It is thought to have a high impact upon the mother's health as well as the family's functioning and the child's development. Socio-demographic, psych-social, and physical activity factors may all contribute to postpartum mood and ability to cope with responsibilities. The primary aim of this study was to determine which of these factors predicted PND in postpartum women. A secondary aim was to identify the socio-demographic and psycho-social predictors of physical activity in postpartum women.

**Methods:** The study used a cross-sectional correlational design. A sample of 150 postpartum women was sent a package of six standardized questionnaires.

**Results:** There was no association between physical activity and PND; however, older mothers, mothers of younger children, mothers who are less reluctant to ask for help, and mothers who are more satisfied with the help they get experience lower levels of PND. Mothers of older babies, mothers with more children, and less educated mothers are more likely to engage in caregiving activities, whereas mothers with fewer children and higher levels of partner support are more likely to engage in occupational activities. None of the socio-demographic factors or any of the parenting factors predicted levels of sporting activity.

## Introduction

Approximately 13 per cent of childbearing women around the world suffer from depression (Gale and Harlow, 2003; Robertson et al., 2004; Grussu and Quantraro, 2009). Maternal perinatal depression, that is depression occurring during pregnancy and in the 6 months following the birth of the child, can have profound effects upon the health and well-being of the mother, her baby and the family (Meltzer-Brody, 2011). This distressing condition is one of the most common complications in both the prenatal and postnatal period and has a prevalence of 10 to 15 per cent in women of childbearing age (Gavin et al., 2005; Gaynes et al., 2005). Depression occurring in women in the postnatal or postpartum period, commonly called postnatal depression (PND), has been a focus of research in high-income countries (O'Hara and Swain, 1996; Robertson et al., 2004; Gavin et al., 2005) and more recently in low- and middle-income countries (Parsons et al., 2012). As socio-economic status is a key moderator of the effects of PND on parenting problems and subsequent child development (Stein et al., 2008), findings in relation to income are important. A review of prevalence studies indicates that rates of PND are significantly higher in low- and middle-income countries than in high-income countries (Parsons et al., 2012).

### Postnatal depression and associated factors

Not only is PND distressing for the mother, it also has the capacity to affect their mothering role and function (Boyce, 2003; Brockington, 2004; Gaynes et al., 2005; Hatton et al., 2005). Previous studies have considered a range of associated socio-demographic and psychosocial factors that may either predict or affect PND. Unfortunately, the vast majority have been limited due to small sample sizes (Blum et al., 2004; Özbaoaran et al., 2011). Nevertheless, some important information has emerged. As noted, low income is a predictor of PND (Patel et al., 2002; Hamdan et al., 2008; Parsons et al., 2012). It has also been suggested that level of education (Singh-Manoux et al., 2002; Araya et al., 2003; Artazcoz et al., 2004; Robertson et al., 2005; Grussu and Quantraro, 2009), employment status (Ghubash and Eapen, 2009; Hamdan and Tamim, 2011), and mothers' age (Cooper et al., 1999; Rubertsson et al., 2003) are associated with PND, although some research contradicts these findings (Leigh and Milgrom, 2008).

Psychosocial factors have been a focus of research, for example by evaluating the role of partner support and/or social support during the postpartum period (Zoltanick et al., 2001; Seimyr et al., 2004). A number of studies have suggested a good quality relationship (regardless of the source of support) is likely to affect postpartum health (Beck, 1996; O'Hara and Swain, 1996). Similarly, lack of partner support has been found to be a strong predictor of PND (Milgrom et al., 2008). Furthermore, poor parent self-esteem and self-confidence and an inability to cope with childhood responsibilities are thought to place mothers at risk of PND (Leigh and Milgrom, 2008; Ghubash and Eapen, 2009; Vernon et al., 2010). Inconsistencies in the literature prevent a clear understanding of the factors associated with PND, thus further research is required.

### Physical activity and exercise in the postpartum period

There are growing concerns regarding postpartum health, weight retention, breastfeeding, and physical activity; however, available guidelines do not provide sufficient instruction and support for women following the birth of their child (Mottola, 2002). The literature is well-documented in terms of the benefits of aerobic fitness and the outcomes of exercise on the general population (Stein, 2005), and also specifically for pregnant women (Symons Downs and Hausenblas, 2004). To date, little attention has been paid to postpartum lifestyle, with a small number of studies focusing on weight retention (LaCoursiere et al., 2006) and milk supply (Currie, 2004). An even smaller amount of research has been done in regards to the postpartum recovery and care.

One study on postpartum women within 4–38 weeks of childbirth revealed specific exercise facilitated the mother's recovery process (Da Costa et al., 2009). According to research by McIntosh (1993) factors such as lack of support, lack of time, and frequent feeling of isolation could contribute to the mental health and recovery in the postpartum period. Difficulties with motherhood such as inability to breastfeed have been reported from other studies (Cooke, 1996; McVeigh, 1997) and would greatly minimize the effect of physical activity and more specifically structured exercise. Inconsistent results have been reported in this area. This is partly attributable to lack of understanding regarding general physical activity, which may include household and caring tasks, and specific exercise, such as that which occurs by participating in sport, or activities such as cycling and walking.

### Physical activity, exercise and postnatal depression

Overall the association between general physical activity and postpartum general health (Sampselle et al., 1999) and postnatal mood disorder (Bowen and Muhajarine, 2006) is unknown and research in this area is warranted. Within the general population, research has often associated physical activity and exercise with decreases in levels of depression, anxiety and stress (Nabkasorn et al., 2006; Blumenthal et al., 2007; Blumenthal and Lephuong, 2009), as well as enhancing general wellbeing (Cramer et al., 1991). Within the context of PND, the association between PND and exercise has only been investigated by a small number of researchers (Cramer et al., 1991). A lack of methodological rigor is evident in the earlier stages of designing exercise interventions for the management of depression. With the exception of one study (Armstrong and Edwards, 2004) the focus has been on exercise in the general population (Da Costa et al., 2009). Armstrong and Edwards evaluated an exercise intervention vs. social support approach on a sample of Australian postpartum women. Although employing a randomized control design, their sample size was small. The effect of exercise as a non-pharmacological intervention for enhancing recovery of postpartum depression has been evaluated recently and exercise has been offered as a therapeutic option for mothers and their family (Daley et al., 2007). Inconsistency exists in the literature regarding the relationship between exercise and depression; for instance some studies found mood improvement through the enhancement of physical fitness, whereas others concluded being physically fit would not guarantee the alleviation of depression. In addition, there is no established theory to explain why exercise would be effective for PND (Da Costa et al., 2009).

### Study aim

Despite a growing body of evidence, there is still a large degree of uncertainty associated with factors which may influence PND. This study aimed to determine whether socio-demographic factors (age of the baby, age of the mother, annual income, number of children, and level of education), levels of physical activity (in the domains of care-giving, living, occupation, and sport), social support, partner support, and parental confidence are associated with PND. An additional aim of the study was to determine whether the socio-demographic factors, partner support, and parental confidence are associated with levels of physical activity in the four domains.

## Methods

### Research design

A cross-sectional correlation design was used.

### Participants

A sample of 150 postpartum women were involved in the study. To be eligible for the study mothers had to be within 6–52 weeks postpartum, live in the Perth metropolitan region of Western Australia, not use a wheel chair or have a physical/medical problem that prevents undertaking moderate to vigorous physical activity, be able to read or respond in English, and not have a baby with disabilities or a premature baby of less than 35 weeks gestation.

### Measures

#### The postpartum lifestyle questionnaire

The Postpartum Lifestyle Questionnaire (PLQ) is a 14-page instrument designed to collect details on physical activity participation, parenting confidence, social support, barriers of exercising, screening of PND, partner support, and personal demographics including age, ethnicity, education, income, type of accommodation, and occupation. The statement “your postpartum lifestyle” appeared on the front cover of the PLQ in order to make a good connection between participants and research goals. The PLQ is made up of the following scales and surveys:

***The Edinburgh Postnatal Depression Scale*** (EPDS; Cox et al., 1987) is a widely used self-report measure of PND. It consists of 10-items; each item is scored on a four-point scale from 0–3. The measure reported high levels of reliability (α = 0.88) (Cox et al., 1987).
***The Kaiser Physical Activity Survey*** (KPAS; Sternfeld et al., 1999) is a self-report measure and is divided into four domains: household/caregiving (caring for a child or/and a disabled person, house chores, such as washing, cleaning, and gardening); occupational activities (types of job, and the intensity of work); active living habits (walking, watching TV, bike riding); and participation in sport and exercise (favorite sport, the frequency of the exercise, and the exercise intensity). The KPAS contains 75 items and it takes approximately 20 min to complete. The alpha reliability of the KPAS ranges between 0.79–0.91 (Sternfeld et al., 1999).
***The Karitane Parenting Confidence Scale*** (KPCS; Črnčec et al., 2008) is a self-report measure, consists of 15 items designed to assess parenting confidence, perceived parental self-efficacy and skills in relation to infant care. It was developed in Australia and is valid for parents of children between 0 and 12 months of age (Črnčec et al., 2008). It targets parenting stress, depression and satisfaction following the birth of the baby and has demonstrated expected correlation with self-efficacy, child development, and support (Črnčec et al., 2008). A Cronbach's alpha of 0.81 has been reported for the measure (Črnčec et al., 2008).
***The Dyadic Adjustment Scale*** (DAS-7; Spainer, 1976) exists in two different versions, a 7- item short form and a 32-item long form. It has been suggested that the DAS-7 is better for surveys, interventions, and clinical screening (Sharpley and Cross, 1982; Schumm et al., 1985, 1986). This study used the DAS-7 because it takes less time for participants to complete. Six of the DAS-7 items are rated on a 5-point Likert scale from 0 (complete disagreement or dissatisfaction) to 7 (agreed or satisfied). The internal consistency of the DAS-7 is well established and the Cronbach's alpha is 0.91 (Hunsley et al., 2001). It measures marital adjustment, level of satisfaction, and level of happiness and cooperation.
***The Social Support Interview*** (SSI; O'Hara, 1995) is a self- report measure of social support. It contains three sections: The first section has four items and is designed to determine whether the respondent has a spouse or partner and also to identify the source of support available (confidant, parent, and spouse). Section 2 contains nine items with three sub-scales to each question. Section 3 contains four questions and covers the overall network of support. The item responses range from one (never) to five (always), with the first three questions relating to topics such as turning to someone in times of need, and having access to available sources of instrumental and emotional support. The other question asks about the degree of satisfaction with overall support and whether they feel hesitant to use the available source of support. These scores are then totaled to provide an overall satisfaction with available support score (out of 20). The higher the score, the higher the overall support received. Test-retest reliability was adequate, and three subscales demonstrated adequate internal consistency with alphas ranging from 0.77 to 0.85 (O'Hara, 1995).

A demographic questionnaire included questions about the demographic characteristics of the mother, including level of education, marital relationship, number of children, and measures of socioeconomic status.

### Procedure

The research received approval from the two governing bodies (Curtin University of Technology, and Department of Health Child and Adolescent Health Service) and data collection commenced in March 2010 and concluded in July 2010. Six hundred questionnaires were distributed in the metropolitan areas of Perth (Western Australia) to child health clinics.

The PLQ was distributed in the form of a package that contained the participant's information sheet and a reply paid envelope for the participants. In addition, nurses were provided with an instruction package that included a checklist, advertisement sheet and another reply paid envelope so they could return details of the number of questionnaires distributed to participants (mothers in the 6–52 weeks postpartum period during the time of participation). Postpartum women recruited to the study by the metropolitan child health clinics, and provided active consent before being given the PLQ package.

## Results

The means and standard deviations for the key variables are listed in Table 1 below:

**Table 1:** **Descriptive statistics for the study variables (*N* = 150)**.

	**Mean**	**Std. deviation**
**EPDS**	4.92	3.80
**SOCIAL SUPPORT VARIABLES**		
Rely on help	4.63	0.74
Emotional support	4.61	0.62
Talk about problems	1.52	0.64
Feeling free to talk	1.31	0.60
Needing him	1.45	0.60
Rely on for childcare	2.10	1.12
Feel someone there to help	1.43	0.71
Reluctant to ask for help	3.36	1.07
Generally do ask for help	1.85	0.75
Satisfaction with help	1.39	0.75
**PARENTING VARIABLES**		
Partner support	2.76	0.51
Parental confidence	3.99	0.35
**PHYSICAL ACTIVITY VARIABLES**		
Caregiving	3.03	0.48
Living	2.89	0.50
Occupation	2.76	0.44
Sport	3.07	1.32

SPSS version 20 was used to test two hierarchical multiple regression models. Our primary research goal was to examine the relationships between PND and 21 potential predictors. We partitioned the predictors into socio-demographic variables (age of baby, age of mother, annual income, number of children, and level of education), social support variables (rely on help, emotional support, talk about problems, feeling free to talk, needing him, rely on for childcare, feel someone there to help, reluctant to ask for help, generally do ask for help, and satisfaction with help), parenting variables (partner support, parental confidence), and physical activity variables (care-giving, living, occupation, sport). Our original intention was to test a hierarchical linear regression model in which the socio-demographic predictors were entered on Step 1, the social support predictors on Step 2, the parenting predictors on Step 3, and the physical activity predictors on Step 4. Inspection of Pearson and Spearman correlations, however, concurred that two of the socio-demographic predictors (number of children, level of education) and all four physical activity predictors (care-giving, living, occupation, sport) were not significantly correlated with PND. These predictors were therefore dropped from the regression model. We then tested a three-step hierarchical regression model in which a reduced set of socio-demographic predictors were entered on Step 1 (age of baby, age of mother, annual income), the social support predictors on Step 2, and the parenting predictors on Step 3. The scatterplot of standardized predicted values against standardized studentized residuals showed no evidence for violations of the regression assumptions of linearity, normality, and homoscedasticity; Cook's Distance values were all less than one indicating that no participant had a disproportionate impact on the regression solution; and tolerance values ranged between 0.463 and 0.989 indicating that multicolinearity was not a problem. The results of the regression analysis are reported in Table 2.

**Table 2:** **Hierarchical multiple regression analysis predicting postnatal depression from socio-demographic, social support, and parenting variables (*N* = 140)**.

**Predictors**	***B* [95% CI]**	β	**Squared part correlation (*sr*^2^)**	***p*-value**
**STEP 1**				
Baby age	0.067 [0.010, 0.123]	0.189	0.035	0.021^*^
Mother age	−0.210 [−0.345, −0.076]	−0.256	0.062	0.002^**^
Annual income	−0.446 [−1.093, 0.201]	−0.113	0.012	0.175
*R*^2^ = 0.120^**^				
Adjusted *R*^2^ = 0.100				
**STEP 2**				
Rely on help	0.545 [−0.509, 1.599]	0.104	0.005	0.308
Emotional support	−0.324 [−1.499, 0.851]	−0.052	0.001	0.586
Talk about problems	0.785 [−0.203, 1.773]	0.130	0.012	0.118
Feeling free to talk	0.223 [−0.945, 1.391]	0.034	0.001	0.706
Needing him	0.795 [−0.382, 1.973]	0.124	0.009	0.184
Rely on for childcare	0.327 [−0.246, 0.900]	0.0096	0.006	0.261
Someone there to help	0.298 [−0.625, 1.222]	0.055	0.002	0.524
Reluctant to ask for help	−1.001 [−1.616, −0.387]	−0.276	0.050	0.002^**^
Generally do ask for help	−0.375 [−1.329, 0.579]	−0.073	0.001	0.438
Satisfaction with help	0.900 [0.041, 1.759]	0.178	0.021	0.040^*^
*R*^2^ = 0.393^***^				
▲ *R*^2^ = 0.274^***^				
Adjusted *R*^2^ = 0.331				
**STEP 3**				
Partner support	−0.307 [−1.606, 0.992]	−0.040	0.001	0.641
Parental confidence	−0.398 [−0.228, −0.403]	−0.349	0.088	0.000^***^
*R*^2^ = 0.491^***^				
▲ *R*^2^ = 0.098^***^				
Adjusted *R*^2^ = 0.430				

* *p < 0.05*,

** *p < 0.01*,

*** *p < 0.001*.

Our secondary research goal was to examine the relationships between levels of physical activity and seven potential predictors. We partitioned the predictors into socio-demographic variables (age of baby, age of mother, annual income, number of children, and level of education) and parenting variables (partner support, parental confidence). Our original intention was to test four hierarchical linear regression models—one for each of the four domains of physical activity, namely, living, sport, caregiving, and occupation. In all models, the socio-demographic predictors were to be entered on Step 1 and the parenting predictors on Step 2. Inspection of Pearson and Spearman correlations, however, concurred that not all of the seven predictors were correlated with all of the activity domains. No predictors were correlated with sport. Only one predictor (age of baby) was correlated with living (*r* = 0.188, *p* = 0.022) indicating that mothers of younger children engaged in lower levels of physical activity in the living domain compared to mothers of older children. Four predictors were correlated with caregiving (age of baby, number of children, level of education, and parental confidence) and two with occupation (number of children, partner support). Multiple regression analyses were therefore conducted for caregiving and occupation. For both regression models: The scatterplot of standardized predicted values against standardized studentized residuals showed no evidence for violations of the regression assumptions of linearity, normality, and homoscedasticity; Cook's Distance values were all less than one indicating that no participant had a disproportionate impact on the regression solution; and tolerance values ranged between 0.923 and 0.991 indicating that multicolinearity was not a problem. The results of the regression analyses are reported in Table 3.

**Table 3:** **Multiple regression analyses predicting (1) physical activity in the caregiving domain and (2) physical activity in the occupation domain from socio-demographic and parenting variables (*N* = 147)**.

	***B* [95% CI]**	β	**Squared part correlation (*sr*^2^)**	***p*-value**
**DV: CAREGIVING**				
Baby age	0.007 [0.000, 0.014]	0.169	0.027	0.035^*^
Number of children	0.151 [0.043, 0.260]	0.216	0.045	0.007^**^
Level of education	−0.055 [−0.102, −0.008]	−0.178	0.031	0.023^*^
Parental confidence	0.020 [−0.002, 0.041]	0.143	0.019	0.078
*R*^2^ = 0.157^***^				
Adjusted *R*^2^ = 0.133				
**DV: OCCUPATION**				
Number of children	−0.227 [−0.323, −0.130]	−0.351	0.122	0.000^***^
Partner support	0.162 [−0.035, −0.290]	0.191	0.036	0.013^*^
*R*^2^ = 0.175^***^				
Adjusted *R*^2^ = 0.163				

* *p < 0.05*,

** *p < 0.01*,

*** *p < 0.001*.

## Discussion

The findings suggest that the mother's age, and the baby's age are predictors of PND; the older the mothers, the lower their levels of PND. This could be potentially attributed to increased levels of maturity and life experience. Older mothers may be able to cope with the emotions associated with motherhood more so than younger women. The younger the mothers' babies the lower the level of PND which could be due to the accumulated burden of parenting responsibility possibly leading gradually to PND. These findings are consistent with other studies (O'Hara and Swain, 1996; Rubertsson et al., 2003; Vernon et al., 2010).

In this study, there were no associations between PND and the level of education, which was also noted by Grussu and Quantraro (2009) and Özbaoaran et al. (2011). Conversely, other studies have found education to be a predictor of PND (Araya et al., 2003; Artazcoz et al., 2004; Robertson et al., 2005; Grussu and Quantraro, 2009). Moreover, mothers who had a higher level of education were more at risk of postpartum depression in two relatively recent studies (Ersek and Huber, 2009; Vernon et al., 2010). Further research is needed to clarify the relationship between level of education and PND. There were no associations between PND and the number of children in the current study which is consistent with previous meta-analytic findings (O'Hara and Swain, 1996).

There was no association between PND and physical activity in the current study, which supports findings by Poudevigne and O'Connor (2005) who failed to find an association between physical activity and depressive symptoms in pregnant women. In contrast, Urizar et al. (2005) found that mothers who suffer from anxiety and stress disorders participated less during a 1- to 10-week exercise intervention in contrast to mothers who reported lower levels of anxiety and stress. Moreover, moderate exercise appeared to benefit women diagnosed with PND (National Institute for Health and Clinical Excellence, 2007). Several other studies have shown a therapeutic effect of exercise on PND (Boury et al., 2004; Daley et al., 2007; Daley, 2008; Craig and Howard, 2009) and the mother's mood (Galper et al., 2006; Daley, 2008; stbye, Swamy and Østbye, 2008). These findings are in contrast to the present study's findings. Participants in the current study, however, engaged in very low levels of physical activity. Because of the positive mental and physical health benefits that have been associated with exercise in postpartum women, it is important to identify the factors that maintain physical activity at low levels for this population. This was our secondary research aim; the results associated with this aim are discussed later.

The mothers' reluctance to ask for help and their satisfaction with help were significant predictors (positive and negative, respectively) of PND severity. These results highlight the role of social support in predicting PND and are consistent with findings which establish PND as a reason for poor quality interaction with others (Honey et al., 2003; Milgrom et al., 2008). The presence of social support as a contributor to managing PND has been identified in previous studies (Beck, 2001; Robertson et al., 2004). In addition, it has been claimed that lack of social support and an active network could increase the risk of PND (O'Hara, 1997). Social support has been identified in different contexts by researchers who suggested that it has buffering effects; thus an under developed social network could result in a higher level of depression and emotional conflict (Robertson et al., 2004).

Parental confidence was found to predict PND in the current study. Less parental confidence resulted in higher levels of PND. This could be due to feelings of isolation and lack of self-efficacy following childbirth. In regard to parent confidence and vulnerability to PND, studies have documented that being capable of understanding the baby and the ability to cope and manage motherhood responsibilities will protect mothers from the risk of having PND (Leigh and Milgrom, 2008; Ghubash and Eapen, 2009). In addition lack of confidence has been shown to increase the risk of PND following childbirth (Vernon et al., 2010); these results support the study findings. In general, the quality of the marital relationship has been found to be an indicator of depression following the birth of a child (Beck, 1996; O'Hara and Swain, 1996). Other studies have shown partner support to be a strong predictor of PND (Özbaoaran et al., 2011). In contrast, the present study found no association between partner support and PND.

Contrary to expectations, exercise was not significantly associated with the level of PND in the present study. This result might reflect a restricted range of scores on the KPAS resulting from the low levels of physical activity reported by this sample (Table 3). The finding supports the study by Demissie et al. (2011) who failed to establish any correlation between recreational activities and depressive symptoms in postpartum women. The results demonstrated that there was no association between being at risk of PND and exercise participation. However, as the effects of exercise on mood have been confirmed frequently in the general population and given that exercise is good for physical and psychological general health, further analyses were conducted on the predictors of physical activity in order to identify potential facilitators of exercise in postpartum women.

None of the socio-demographic factors (age of baby, age of mother, annual income, number of children, and level of education) or any of the parenting factors (partner support, parental confidence) predicted levels of sporting activity.

Mothers with older babies were more active in the caregiving, occupation, and living domains. These findings are consistent with those of Sampselle et al. (1999) which show that children's age can affect physical activity in the postpartum period. The present finding that mothers of older babies were more active in all three domains, however, is somewhat counter-intuitive in that mothers with older babies might be expected to becoming *less* active in the caregiving domain with a consequent increase in activity in the living and occupation domains. Not surprisingly, mothers with more children were more active in the care-giving domain and less active in the occupation domains.

Less highly educated mothers were more active in the care-giving domain, which might be due to their inability to find well-paid employment—a state of affairs that would lessen the incentive to work outside of the home. This idea is supported by studies that have found a link between income and physical activity in the postpartum period (e.g., Eyler et al., 2002). In general, however, the links between education and physical activity, and between income and physical activity, are not well-established. Young et al. (1998) failed to find an association between education level and physical activity; and Bild et al. (1993) and Ransdell and Wells (1998) failed to find an association between income and physical activity. Our failure to find an association between income and physical activity might reflect the fact that most of our participants were in higher income brackets.

There was no association between parental confidence and physical activity in any of the four domains. Our failure to find a link between parental confidence and activity in the occupation domain is inconsistent with the finding that feeling more confident and able to cope with maternal roles was associated with less participation in occupational activities (Blum et al., 2004). We also found that mothers with higher levels of partner support were more active in the occupation domain, which is not inconsistent with findings from other studies showing that partner support makes it easier for mothers to engage in physical activity after child birth (Mottola and Campbell, 2003; Blum et al., 2004; Albright et al., 2006; Symons Downs and Ulbrecht, 2006; Kanotra et al., 2007).

There were some limitations for the current study such as the size of the sample, a lack of clinically depressed mothers in the sample and the duration of the study. Increasing the size of the sample, thus ensuring greater statistical power, might result in the capture of associations that the current study missed and resolve some of the disagreement between different studies. In addition, the inclusion of greater numbers of clinically depressed mothers in the study, while methodologically challenging, would strengthen the findings and perhaps add statistical support for the hypothesis that exercise can help to mediate the effects of PND.

## Conclusion

The study findings suggest that there is no association between physical activity participation PND following childbirth; however, older mothers, mothers of younger children, mothers who are less reluctant to ask for help, and mothers who are more satisfied with the help they get, experience lower levels of PND. In addition, mothers of older babies, mothers with more children, and less educated mothers are more likely to engage in caregiving activities, whereas mothers with fewer children higher levels of partner support are more likely to engage in occupational activities. Further research is needed in order to further investigate the relationship between exercise and postpartum mood. The factors that enable exercise need to be investigated during pregnancy and the prenatal period to facilitate postnatal exercise and encourage more participation in postnatal exercise classes, as women appear to be mostly inactive during this time, possibly due to adapting to their new role and responsibilities. In addition, qualitative approaches are required to inform the development of structured exercise programs that are tailored to new mothers' specific needs and maintain their participation.

### Conflict of interest statement

The authors declare that the research was conducted in the absence of any commercial or financial relationships that could be construed as a potential conflict of interest.
